# Novel alternatively-spliced exons of the VRK2 gene in mouse brain and microglial cells

**DOI:** 10.1007/s11033-020-05584-3

**Published:** 2020-06-24

**Authors:** Salsabil Almarzooq, Jaedeok Kwon, Ashleigh Willis, John Craig, Brian J. Morris

**Affiliations:** Institute of Neuroscience and Psychology, College of Medical, Veterinary and Life Sciences, University of Glasgow, Sir James Black Building, G12 8QQ Glasgow, UK

**Keywords:** Schizophrenia, Risk gene, Splice variants, Truncated isoform

## Abstract

Common sequence variations in the *VRK2* gene contribute to genetic risk for various psychiatric diseases including schizophrenia and major depressive disorder. Despite the clear importance of studying the regulation and function of VRK2 for understanding the causes of these diseases, the organisation and expression of the gene remain poorly characterised. Using reverse-transcriptase-PCR, we have amplifed exons of *Vrk2* mRNA from regions of mouse brain, and from different cell classes comprising neurones, astrocytes and microglial cells. We find that *Vrk2* mRNA is expressed in all cell types, and that the splicing of the mouse *Vrk2* gene is much more complex than previously appreciated. In addition to the predicted alternative splicing (absence/presence) of the penultimate 3 prime exon, we also detected a variety of 5 prime structures, including two novel exons spanning the first characterised exon (exon 1), which we term exons 1a and 1b. While expressed in neurones and astrocytes, exon 1b was not expressed in microglial cells. Expression of transcripts containing exon 1a in microglia was increased by immune stimulation. An additional truncated transcript lacking 7 central exons was also identified. As with the human gene, the results confirm complex patterns of alternative splicing which are likely to be relevant for understanding the physiological and pathological function of the gene in the CNS.

**Electronic supplementary material:**

The online version of this article (10.1007/s11033-020-05584-3) contains supplementary material, which is available to authorized users.

## Introduction

The Type 2 Vaccinia Related Kinase (*VRK2*) gene codes for a serine threonine kinase belonging to the casein kinase 1 group, and is situated in the 2p16.1 chromosomal region. VRK2 was initially recognised in highly proliferative cells (e.g. thymus and foetal liver cells) and early theories emphasised a role in cell division and cell cycle regulation [1]. VRK2’s importance is however not limited to proliferating peripheral cells, and it was subsequently found to be also expressed in brain [2]. In fact, sequence variants in the *VRK2* gene are robustly associated with schizophrenia [3–5], and also with depression [6], bipolar disorder [7], and epilepsy [8, 9]. Tesli et al. (2016) [10] demonstrated reduced blood levels of *VRK2* mRNA in a large sample of individuals with schizophrenia relative to controls (p < 10^− 12^), thereby strongly supporting a genetic contribution of VRK2 to the causes of schizophrenia. This result has since been replicated in an independent large sample [11]. It has recently been reported that in the brain, *Vrk2* expression is restricted to microglia [12] If this were verified, this would be of some significance for understanding the causes of schizophrenia, as it would unequivocally implicate microglial dysfunction in disease aetiology.

**Fig. 1:**
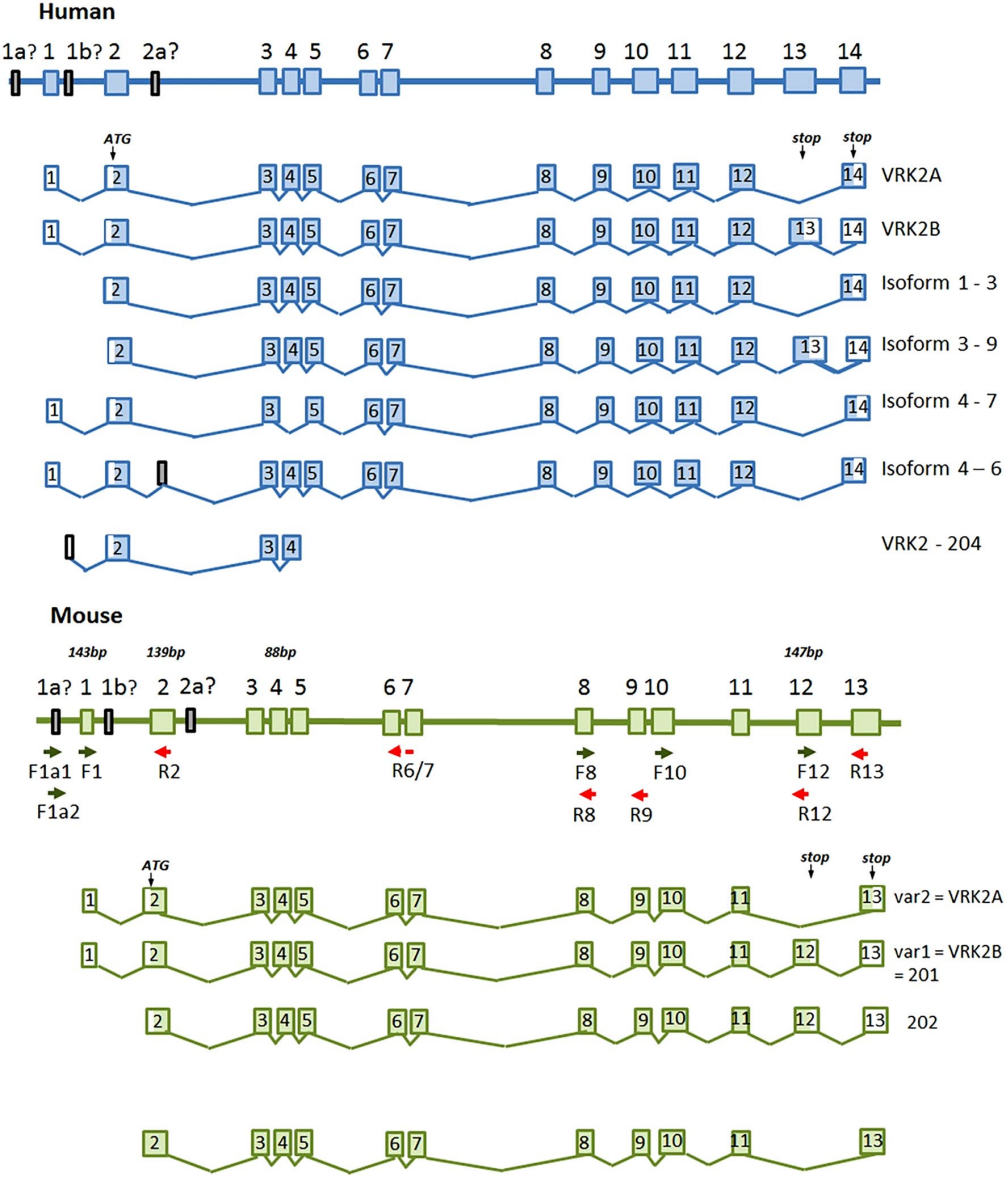
Schematic diagram of intron/exon structure for human (upper) and mouse(lower) VRK2 genes, from 5’ end (left) to 3’end (right). Isoform designations provided are according to RefSeq annotations. Relative lengths of exons/introns are for illustrative purposes, and are only very roughly proportional to actual distances. Approximate locations of PCR primers used in this study are also shown

Blanco et al. [13] identified two isoforms of human VRK2 that have a kinase domain in common, but differing subcellular locations due to distinct C-termini. Thus, VRK2A (where the penultimate 3 prime exon - exon 13 - is absent)(Fig. 1) encodes a 508 amino acid protein which has a transmembrane domain and is localised in the endoplasmic reticulum and mitochondrial membrane. In contrast, VRK2B (where exon 13 is present) encodes a shorter 397 amino acid protein, due to the presence of an earlier stop codon, lacks this domain, and is present in the cytoplasm and the nucleus. While VRK2A is significantly expressed in all human cell lines, expression of VRK2B is more limited and occurs only in certain cell lines. Blanco et al. [14] further noted that the stress responses to cytokines such as interleukin 1β (IL-1β) and to hypoxia are regulated by the more abundant VRK2A. VRK2 forms a complex with JIP1 [15], which functions as a scaffold protein for the c-Jun N-terminal kinase (JNK) pathway by building mitogen-activated protein kinase (MAPK) complexes. This signal transmission route is reportedly blocked by VRK2A, thus limiting the phosphorylation of JNK and AP1-dependent transcription, and hence transmission of this stress signal. By contrast, the VRK2B isoform in the nucleus can reduce the transcriptional response to the inflammatory cytokine interleukin-1β [15].

Examination of Genbank and the European Nucleotide Archive databases suggests the existence of further alternative splicing in human (Fig. 1), mainly focussed around the 5’ end of the mRNA. The mouse Vrk2 gene is recognised as having 13 rather than 14 exons [16], and one of the annotated REFSEQ transcripts lacks the penultimate exon (exon 12)(Fig. 1). However, some of the transcripts predicted by automated genome sequence scanning suggest that there may be further transcript diversity in the mouse, as in the human. Since future studies on *Vrk2* gene function will be needed to explore the genetic basis of psychiatric disease, an understanding of gene structure is a necessary first step. We therefore undertook an investigation of the extent of alternative splicing in mouse brain, and in different cell types within the brain, to clarify the structure of expressed *Vrk2* mRNAs, and the effect of cell type and environment on *Vrk2* gene expression.

## Materials and methods

### Culture of primary cells - preparation of neurons and astrocytes

Primary cerebrocortical neuronal cultures were prepared using brain tissue from wild-type C57BL/6J mouse embryos (E17) as previously described [17–19], and maintained for 12–14 dayd in vitro (DIV). For primary astrocyte cultures [20], the medium was changed every 2 to 3 days, and then, to deplete as many microglia as possible, the flask was shaken at 100 rpm for 30 min on an orbital shaker. After confluency was reached, trypsin-EDTA and HEPES (final concentration 50 mM) were added until the astrocyte containing cell layer slowly detached from the culture flask, while the microglia stayed attached.

The quality and purity of neuronal and astrocyte cultures has been repeatedly checked – cultures are in good health and are around 90% specific for the defined cell type [17–19], as is standard for these techniques.

### Culture of cells - preparation of microglia cells

SIM-A9 cells (ATCC, #CRL-3265), which are a spontaneously immortalized mouse microglial cell line [21, 22], were were maintained in DMEM/F12 medium serum (Invitogen, Paisley, UK) supplemented with 5% horse serum (Invitogen, Paisley, UK) and 1% penicillin-streptomycin serum (Invitogen, Paisley, UK), at 37 °C. Cells were used at passage 12–15. The culture medium was replaced with DMEM/F12 medium without horse serum at 50 to 60% confluence overnight prior to the indicated treatment ( 100 ng/mL LPS or vehicle control) for 24 h.

### RNA isolation

#### RNA isolation from cells

Total RNA was isolated from cells by removing the medium from the wells, and the cells being washed with phosphate-buffered saline (D-PBS, Invitrogen). The cells were then detached from the well surface using 0.05% trypsin and inactivated by the addition of 0.5 ml DMEM. The detached cells were transferred to a tube and pelleted by centrifugation at 10,000 rpm for 5 min at room temperature. Each cell pellet was washed with D-PBS and centrifuged again at 10,000 rpm for 5 min at room temperature. Following this, an RNeasy mini kit (Qiagen, #74104) was used to isolate total RNA from the cells as described above.

#### RNA isolation from mouse brain tissues

Total RNA was isolated from mouse C57BL/6J brain tissue using the RNeasy mini kit (Qiagen, #74104) according to the manufacturer’s protocols, and as previously described [23, 24]. Brain tissues were removed from the RNAlater and placed in ribolyser tubes on ice (Lysing matrix D, BIO 101 Systems, cat. No. 6913 − 100), being subject to mechanical disruption for 20 s at 6.5 g using Hybaid Ribolyser. Following this, an equal volume of 70% ethanol was added to each sample, and the resulting mixes were transferred to an RNeasy column and centrifuged at 10,000 rpm for 15 s at room temperature. The total RNA in the columns then underwent multiple wash steps, including a DNA removal step with an incubation period of 15 min at room temperature. Total RNA was eluted from the columns and the concentration of the isolated RNA checked using a NanoDrop™ 1000 (Thermo Scientific).

### Reverse transcription and PCR

#### cDNA synthesis

The reverse transcriptase step was performed using a high-capacity RNA to cDNA kit (Thermo fisher scientific, #4387406) according to manufacturer’s instructions. For each reaction, 500 ng to 2 µg of total RNA was used per 20µL reaction. First strand cDNA was generated by incubating the tube contents at 37 °C for 60 min, then terminating the reaction at 95°C for 5 min. The quantity of cDNA produced was checked using NanoDrop™ 1000 (Thermo Scientific). Following this, samples were diluted at a 1:5 ratio and stored at −20 °C for further processing.

#### Polymerase chain reaction

Several 10 µl PCR reactions were set up using 0.3 µl of suitable forward and reverse primers, plus 2 µl of cDNA, 0.5 µl of DMSO and 5 µl of Platinum™ Taq Green Hot Start (Invitrogen, # 11966034). No template controls were included. The standard PCR reaction conditions were 3 min at 95 °C and then 40 cycles of 30 s at 95 °C, 30 s at 60 °C, and 1 min at 72 °C. The latter step was modified to 90 s when the expected product was greater than 1,000 bp. The final elongation step was at 72 °C for 7 min. Primer sequences are provided in Supplementary Table 1.

#### Gel electrophoresis and visualisation of amplicons

PCR products were separated out by gel electrophoresis in a 2% agarose gel made of UltraPure™ Agarose (Invitrogen, #16500-500). The gel was stained with ethidium bromide (Sigma, #E1510), with 1 µl of ethidium bromide added to each 300 ml of melted agarose. Then, 6 µl of the 100 bp DNA ladder (NEB, #N3231S) and 8 µl of each PCR sample was loaded into each well and electrophoresis was performed at 70 V. Separated samples were visualised by means of ultraviolet light through safety glass/visors as well as being photographed.

#### Amplicon sequencing

The desired PCR products were carefully cut out of the gel and the DNA fragments extracted and purified using a QIAquick Gel Extraction Kit (Qiagen, #28704) per manufacturer’s protocols. A small amount of the purified product was run on a 2% agarose gel to ensure the correct size was maintained. The remaining purified product, with DNA concentration of 20–30 ng/µl ) was sent to the GATC service, (Germany) for sequencing.

## Results

PCR amplicons of various sizes were detected with many intron-spanning primer combinations.

### Exon 12 (penultimate 3’ exon) splicing

**Fig. 2:**
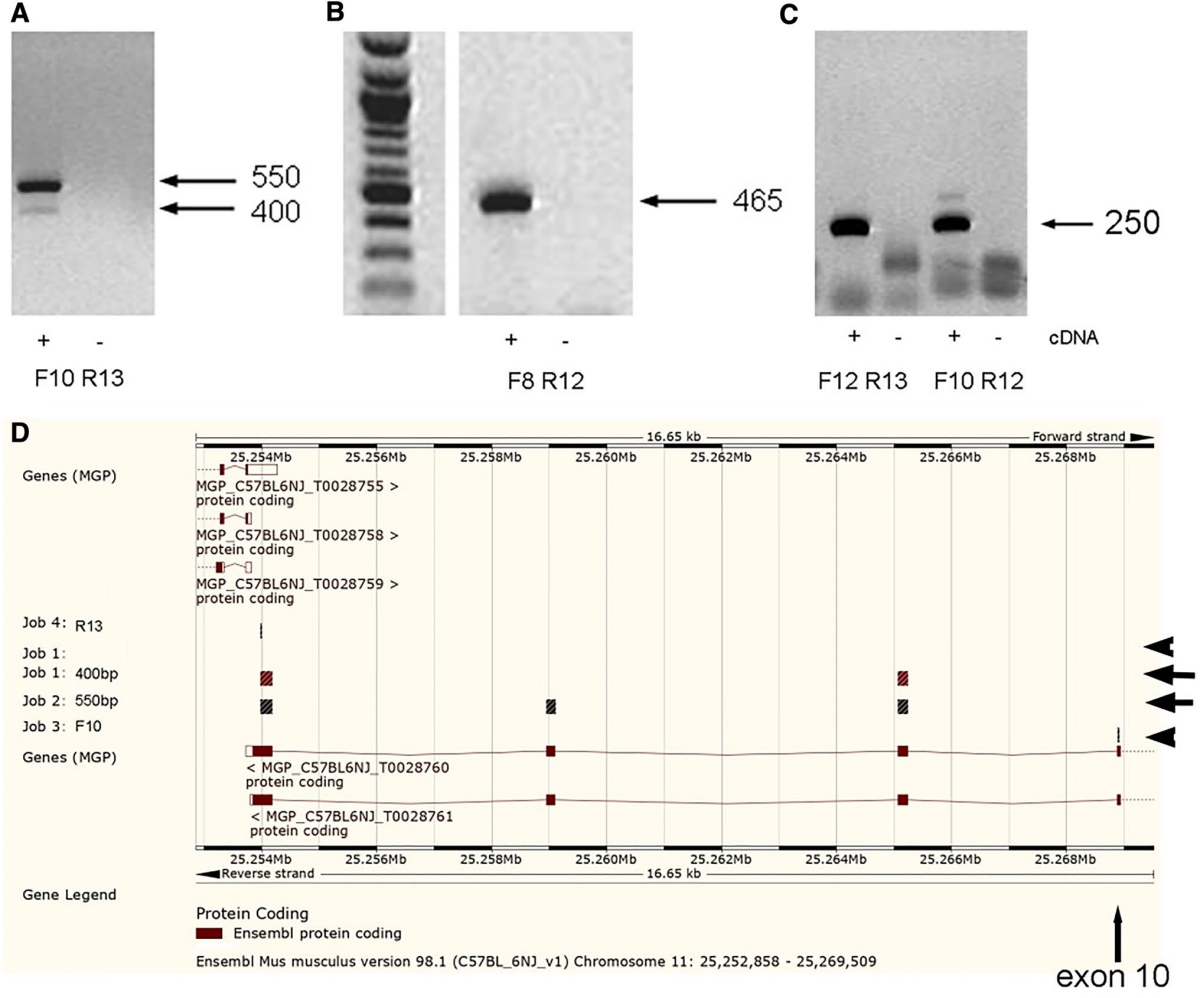
Isoform detection in microglia. cDNA was prepared from microglial cells, and amplified with primers spanning exons 10 to 13 (**a**), 8 to 12 (**b**) and 12 to 13 or 10 to 12 (**c**). Alignment of sequenced amplicons from A (arrows) against the Ensembl genome browser is also shown (**d**), with location of F10 and R13 primers (arrowheads). Exon 12 is 147 bp in length

Amplicons of 2 sizes, differing by ~ 150 bp, were detected in microglial cells with primers from exon 10 to exon 13 (Fig. 2a). The larger amplicon, confirmed by sequencing to contain exon 12, was of notable higher intensity than the amplicon confirmed by sequencing to lack exon 12. Consistent with this, strong amplification was obtained when primers were used that specifically amplified exon 12-containing transcripts (Fig. 2b-c).

**Fig. 3:**
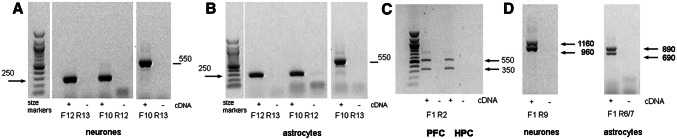
Isoform detection in neurones, astrocytes and microglia. **a**,**b** cDNA prepared from primary neuronal cultures (**a**), or primary astrocyte cultures (**b**), was amplified with primers targetting exons 10–13. In all cases, amplicons are obtained when the primer sequence lies within exon 12. Note that the predicted amplicon for isoforms lacking exon 12 (400 bp with F10 R13) is not detected in neurones or astrocytes. **c**, **d** Transcripts amplified containing exon 1. **c** expression of 5’ exons in adult mouse prefrontal cortex (PFC) and hippocampus (HPC): amplicons were generating using F1 and R2 primers. **d** amplicons generated using F1 and R9 primers in cDNA from cultured neurones, or using F1 and R6/7 primers in cDNA from cultured astrocytes. In each case, 2 bands differing in size by around 200 bp are obtained

Neurones and astrocytes also generated strong amplification with primers placed within exon 12 (Fig. 3a,b). However, only transcripts containing exon 12 could be clearly identified - primers targetting exons 10 and 13 yielded a predominant band of 550 bp, whereas isoforms lacking exon 12 would yield a 400 bp band.

VRK2 has been linked to immune responses (Lee 2019, Blanco et al. (2006). We therefore investigated the effect of immune stimulation, using the bacterial mimetic lipopolysaccharide (LPS), on *Vrk2* transcript expression. While LPS produced a clear inflammatory response in the microglial cells (assessed by iNOS induction, Supplementary Fig. 1), no alteration in the expression of a variety of amplicons was detected (Supplementary Fig. 2).

### 5’ exon splicing

Only a single amplicon of around 350 bp was observed when primers targetting exons 1–2 were employed in the microglial cells (Supplementary Fig. 2). However, when mouse brain tissue was studied, an extra amplicon was observed, which was larger by ~ 200 bp (Fig. 3c), suggesting the presence of an additional exon between exons 1 and 2. This extra size was also detected in longer transcripts from neurons and astrocytes, whatever the position of the reverse primer (Fig. 3d).

**Fig. 4:**
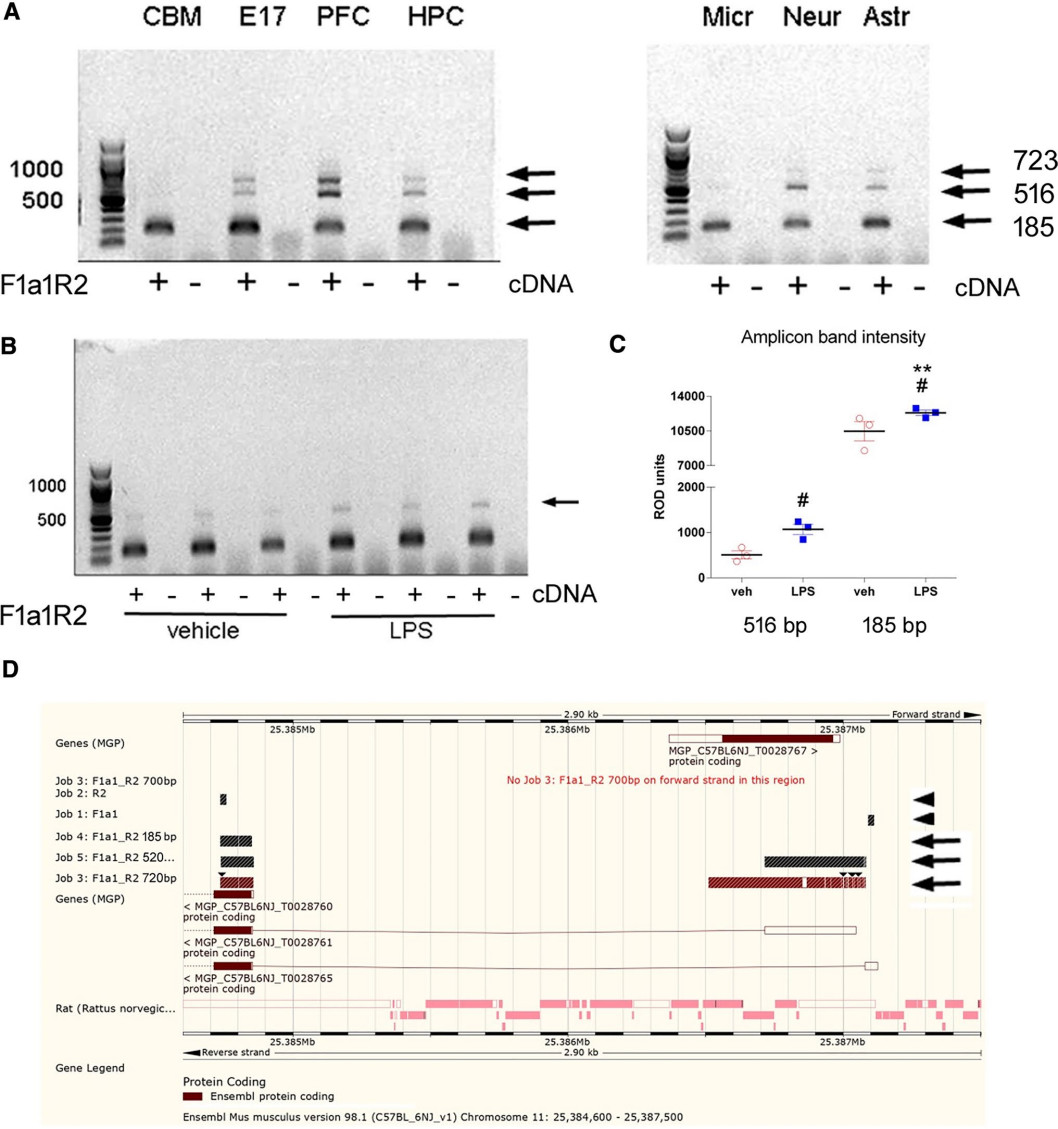
Transcripts amplified containing exon 1a. Expression of 5’ exons including exon 1a in **a** brain tissue (embryonic E17 whole brain, CBM, PFC or HPC) different CNS cell types (microglia, neurones or astrocytes), and **b** microglial cells exposed to either vehicle or LPS (100 ng/ml) for 18 h. **c** Graph shows amplicon band intensity for the larger and smaller bands in C (individual data points with mean +/- s.e.m.). # p < 0.05 vs. corresponding vehicle group; Mann Whitney test ** p = 0.015 vs. corresponding vehicle group (one sided t-test). **d** Location of F1a1 and R2 primers (arrowheads), along with alignment of sequenced amplicons from brain (185 bp, 520 and 720 bp) (arrows) against the Ensembl genome browser. The amplicons represent exons 1a, 1a + 1, and 1a + 1 + 1b, respectively, plus exon 2

We tested an additional forward primer located further upstream from the start of exon 1, to test for the possible presence of an additional 5’ exon. The Ensembl database shows an automated prediction of an exon in this region, derived from a potential *Vrk2* transcript that terminates in exon 4. Using a forward primer designed for this exon (which we term exon 1a), three PCR amplicons were detected with the same reverse primer (exon2), in brain tissue and in cultured neurones and astrocytes (Fig. 4a). Microglial cells, and also cerebellar tissue, only showed the presence of the smallest exon 1a-containing amplicon, suggesting that they do not express splice variants containing exons 1 and 1b as well as 1a.

Sequencing of the amplicons revealed that the shortest (185 bp) amplicon has exon 1a spliced directly to exon 2, the middle amplicon contains exon 1a, exon 1 and exon 2, and the longest (723 bp) amplicon contains exon 1a, exon 1, and an additional exon (which we term exon 1b) derived by the lack of a splicing event at the end of exon 1 (Fig. 4d and Supplementary Fig. 3).

If the automated prediction exon start site is presumed correct, then exon 1a is 80 bp in length. Our data show that exon 1b is 205 bp in length. Neither exon is predicted to alter the open reading frame (Supplementary Fig. 3).

Exposure of microglial cells to an immune stimulus (100 ng/ml LPS, 24 h) resulted in an increase in the expression of the exon 1a-containing transcripts (Fig. 4b, c).

**Fig. 5:**
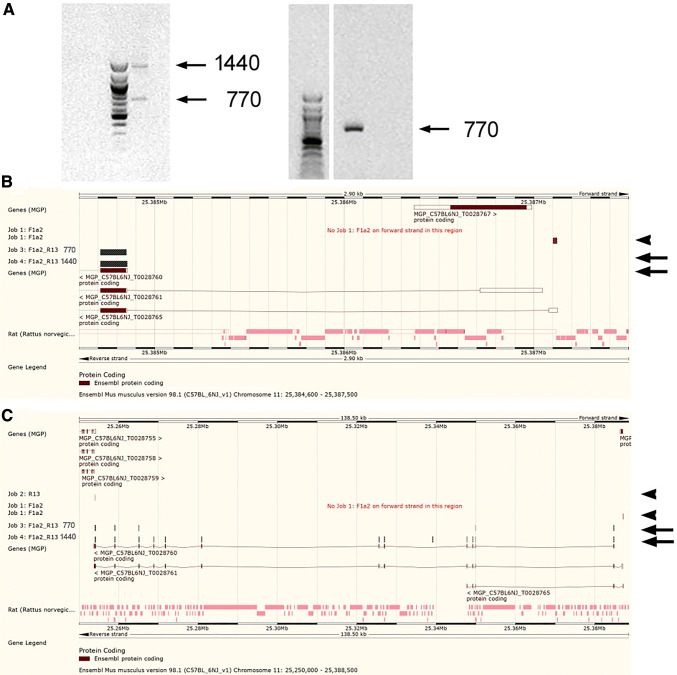
Identification of full length transcripts (exon 1a-13), and also an atypical isoform lacking exons 4–10. **a** Left panel: two major sizes of amplicons were obtained from microglial cells using primers targetting exon 1a and exon 13. Right panel: a single amplicon was obtained from adult mouse prefrontal cortex tissue using primers targetting exon 1 and exon 13. **b**,**c** Alignment of sequenced 1500 bp and 800 bp amplicons (arrows) from microglial cells against the Ensembl genome browser is shown for the 5’ end of the gene **b** and for the full gene **c** (Note that both transcripts lack exon 1). Location of F1a1 and R13 primers is also shown (arrowheads)

Amplicons were identified that stretched from exon 1a through to exon 13. A truncated transcript of around 750 bp was also detected in microglial cells, which was revealed by sequencing to lack exons 4–10 (Fig. 5a-c). A similar short amplicon of around 800 bp was obtained using primers targetting exons 1 and 13 in mouse prefrontal cortex tissue (Fig. 5a).

## Discussion

Most previous studies of VRK2 expression have focussed on proliferating cells, despite the emerging importance of VRK2 for the aetiology of psychiatric disease. To test for cell-type-specific expression in the CNS, we analysed RNA extracts from cultured neurons and astrocytes, and from a cultured mouse microglial cell line. Unexpectedly, Vrk2 seems to be expressed in all three cell types. Lee et al. [12] reported, using RT-PCR targetting exon 13, that *Vrk2* expression in the mouse CNS is confined to microglia. The reasons for the discrepancy with our study are not clear. While we have not definitively characterised the 3’ end of all the splice variants reported here, amplicons containing exon 13 did not show preferential expression in microglial cells.

We confirm the existence, in the mouse, of homologues of the human VRK2A and VRK2B isoforms, lacking and containing the penultimate 3’ exon. We also report additional complexity in the alternative splicing of other exons of the mouse *Vrk2* gene. Interestingly, the expression pattern for the various isoforms is highly variable among the different cell type. For example, transcripts containing the newly-identified exon 1a do not also contain exon 1 in microglia, whereas longer exon 1a-containing transcripts are also found in neurones and astrocytes. Interestingly, of the exon 1a-containing isoforms, the shortest (exon 1 and 1b-lacking) transcripts appear to predominate in all cell types.

The heterogeneity of 5’ utr variants is remarkable. We confirmed that full length transcripts incorporating exon 1a are expressed (Fig. 5). As far as we can ascertain, none of the novel variants extends the ORF, hence leaving the N-terminal protein sequence unaffected. The functional significance, of a process that is clearly regulated by cell type and environment, remains to be determined. Note that the Ensembl database records human VRK2 isoforms with between 3 and 6 additional 5’ exons upstream of the exon conventionally designated “exon 1” by Palo and coworkers, by Niskrat and Taco, and by ourselves. These additional exons are also not predicted to change the ORF. One possibility is that the increasing 5’utr lengths allow increased thermodynamic stability of tertiary structure, thus impeding translation. Indeed RNAfold [25] predicts that the 1a-, 1a-1- and 1a-1-1b- 5’utrs have tertiary structures with negative free energies of 52, 240 and 357 kcal/mol respectively.

Cell samples were also analysed for the presence of an alternative isoform that lacks exon 12. We found this isoform was expressed in microglia, but was not clearly detected in astrocytes or neurons (Fig. 3). The isoform lacking exon 12 (corresponding to VRK2A in human) is the cytoplasmic form, while the longer mRNA VRK2B form is nuclear [14, 15]. It therefore appears that the cytoplasmic function is more relevant for microglial cells in the CNS. It is interesting to note that VRK2A is more abundant than VRK2B in a variety of (highly proliferative) tumour cell lines [13], whereas our data suggest that VRK2B is more abundant in slowly-proliferating cells (microglia and astrocytes) and non-proliferating cells (neurons). We speculate that prominent VRK2A expression may relate to the suggested role of VRK2 in cell proliferation, and that higher relative VRK2B expression may be a feature of non-proliferating cells. We note that the brain tissue used here was derived from male mice, and it should be remembered that sex-differences can play a role in gene expression in brain [26], and hence that female tissue may show differences.

We also detected the presence of a truncated transcript, which sequencing revealed to be missing 7 central exons (exons 4–10). This truncated form produces a predicted protein of only 59 amino acids, where the C-terminal 17 amino acids (QENFESRWSTFRATGIFH) are not present in the longer forms of the protein. The functional significance of this new variant is not clear. It is expressed in microglial cells, but will not have a functional catalytic site, or the JIP-1 binding site [15]. In terms of CNS disease risk, the epilepsy risk mutation identified is within exon 2 [8], while the bipolar disorder mutation is in exon 4 [7]. This transcript is therefore unlikely to contribute to bipolar disorder risk, but may play a role in epilepsy. The precise exon composition of variants expressed in neurones and astrocytes that contain the catalytic site remains to be determined, although they clearly exist (Fig. 3d), and will presumably contain exon 12. However, the data emphasise the differences between *Vrk2* spicing in neurones/astrocytes and microglial cells.

### LPS treatment induces alternative splicing

Vrk2 is known for its role in modulating inflammatory responses, thus one aim of this study was to investigate the correlation between inflammatory status and the distribution of splicing events in LPS stimulated microglial cells. LPS is a well-characterised bacterial mimetic that elicits inflammatory response in the brain. No obvious differences in expression were detected for canonical transcripts (containing exon 1) or for the main transcripts either containing or lacking exon 12 (Supplementary Fig. 2). Intriguingly, transcripts containing exon 1a showed clearly stronger expression in the LPS-treated cells (Fig. 4). This indicates that *Vrk2* splicing pattern is altered by immune stimuli, and suggests some particular immune-response function for exon 1a.

## Conclusions

Overall, our data reveal the existence of novel alternatively-spliced isoforms of VRK2, and indicate that splicing is regulated according to cell type, brain region, and environmental conditions. Further investigations will be required to understand the functional significance of these complex patterns of splicing, and whether similar complexity exists in the human gene.

## Electronic supplementary material

Below is the link to the electronic supplementary material.

Supplementary file1 (PDF 671 kb)
