# Supplementary material for: Novel alternatively-spliced exons of the VRK2 gene in mouse brain and microglial cells
Source: Mol Biol Rep. 2020 Jun 24;47(7):5127–36. doi: 10.1007/s11033-020-05584-3 (PMC7417415; doi:10.1007/s11033-020-05584-3)
Supplement: Supplementary file 1 — Supplementary file1 (PDF 671 kb) [file 11033_2020_5584_MOESM1_ESM.pdf]

Supplementary Information

Novel alternatively-spliced exons of the VRK2 gene in mouse brain and microglial cells

Salsabil Almarzooq, Jaedeok Kwon, Ashleigh Willis, John Craig and Brian J. Morris

Supplementary Table 1, and Supplementary Figures 1-3

Supplementary Table 1,

**Forward primer**	**sequence**	**Reverse primer**	**sequence**
F1a1	TCTGGGTATGCCTAGAGGCTG	R2	TCCAGAGCCGATCATCTTGC
F1a2	TACAAGGCTGTCTGGGTATGC	R6,7	CCAGCATCCTGATGCCAAGT
F1	GTCCGGTGCTTGGAAACTCT	R8	GGCCCTTTCTGGGATCTTCC
F8	AGGAAGATCCCAGAAAGGGC	R9	CAGTGCAGCATGCAGTAACC
F10	GTGCTTAAGTGGGCTCCTTCT	R12	TAGCTGGGAACTCATTGGCTG
F12	AAGACAGCCAGCCCACAATG	R13	GTGACTCCCAGATTACCCGTG

Table 1: Primer sequences used in this study

Supplementary Figure 1

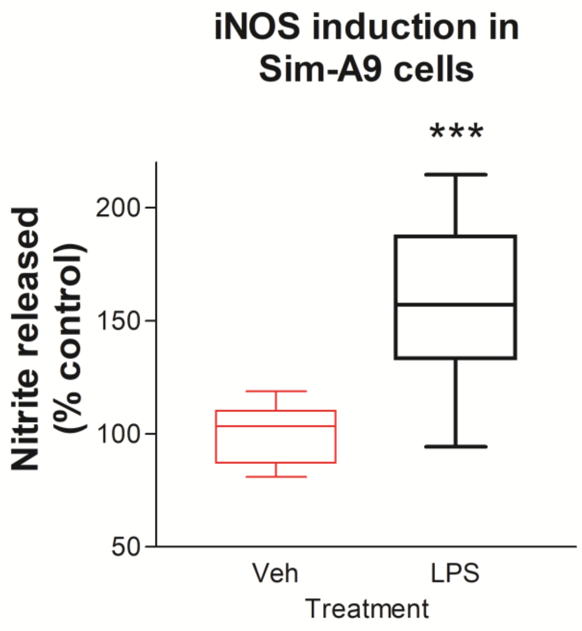

Induction of inducible nitric oxide synthase by LPS in Sim-A9 cells. Cells were exposed to 100ng/ml LPS or vehicle (PBS) for 18h, and then media samples were assessed in triplicate for nitrite content using the Griess assay (Enzo, UK). Results are expressed as the percentage of the mean control (vehicle) value in that experiment, and are as shown as box plots (median with inter-quartile range) and “Tukey” whiskers. n= 12/group,*** p=0.001 vs vehicle group (one sided t-test).

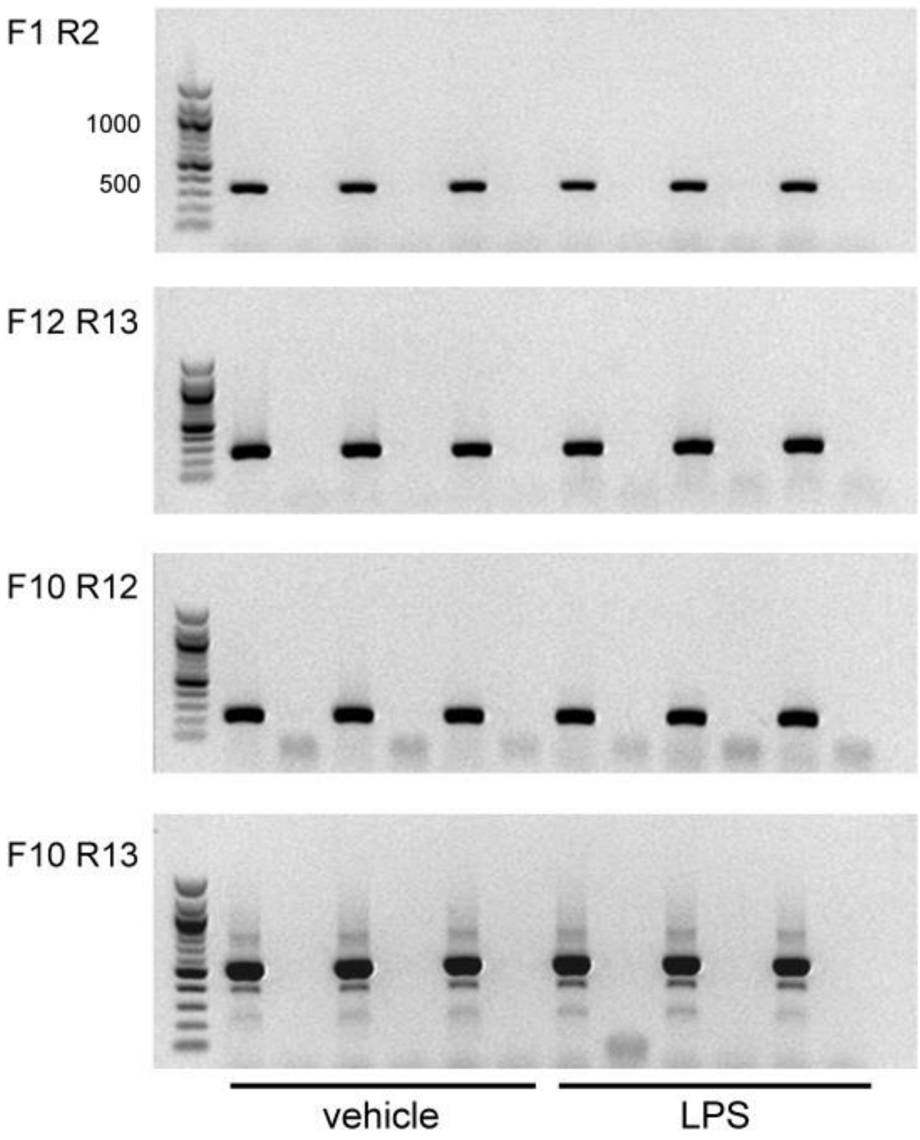

Supplementary Figure 2

Effect of immune stimulation on exon expression in microglial cells. Cells were exposed to vehicle or LPS (100 ng/ml) for 18 h, and then, once RNA was extracted and converted to cDNA, PCR was performed. Primers targetted (A) exon 1 and exon 2; (B) exon 12 and exon 13; (C) exon 10 and exon 12; or (D) exon 10 and exon 13. The left lane has the size markers. Each other lane represents a separate biological sample. Note that for the F10R13 amplification, apart from the specific 550 bp and 400 bp bands, there are some weak, non-specific bands present that are not present in Figure 2.

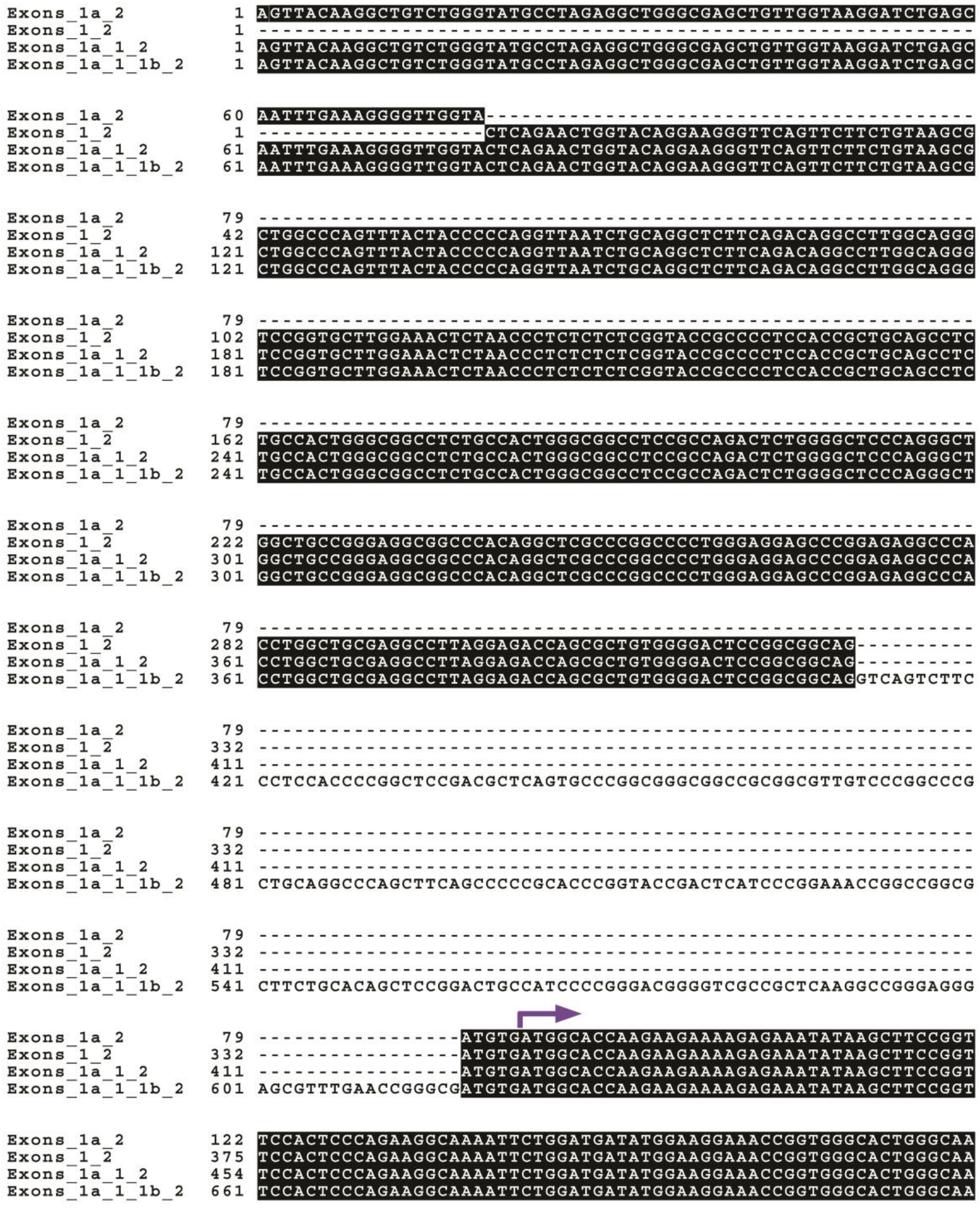

Supplementary Figure 3

Alignment of 5’ sequences, showing variable 5’ utr and the beginning of the ORF (arrow).
